# 

**Published:** 1917-10-27

**Authors:** 


Rates of Subscription: Twelve months, 6/6; Six months, 4/-; three months, 2/-, post free to any address in the United Kingdom. Abroad, Twelve months, 8/8; Six months, 5/-; Three months, 2/6, post free. **THE HOSPITAL "Index" to Volume LXI., October 1916 to March 1917, is now ready, price 2½d. per copy, post free.** Address The Manager, The Hospital, "The Hospital" Building, 28/29 Southampton Street, Strand, London, W.C. 2.

### Matrons' and Sisters' Appointments.

Ottery St. Mary Cottage Hospital.—Miss E. S. Newton has been appointed matron. She was trained at the Great Northern Central Hospital, London, and has since been night sister at Newbury Hospital and sister at the General Hospital, Tiverton.

Cottage Hospital, Ellesmere.—Miss Betha Hansen has been appointed nurse matron. She was trained at the Toxteth Infirmary, Liverpool, and has been charge nurse at the Wallasey Borough Hospital, and surgical charge and theatre sister at the 2nd Birmingham War Hospital.

Northumberland War Hospital, Gosforth.—Miss Grace Mary Nichol has been appointed assistant matron. She was trained at the Nottingham General Hospital, and has been ward sister, temporary night superintendent and theatre sister, and assistant matron at the Cumberland Infirmary, Carlisle.

Royal Infirmary, Preston.—Miss Emily Davis has been appointed sister. She was trained at the Royal Infirmary, Hull, at which institution she has taken sister's holiday duties.

Warrington Infirmary and Dispensary.—Miss M. A. Ford has been appointed home sister. She was trained at the Salford Union Infirmary, Manchester, and has been night sister at the Auxiliary Military Hospital, Frodsham, Cheshire, and ward sister at the Grange Auxiliary Military Hospital, Southport.

Hahnemann Convalescent Home, Bournemouth.—Miss M. T. Foulkes, Q.A.I.M.N.S.R., has been appointed sister. She was trained at the Preston and County of Lancaster Royal Infirmary, afterwards being theatre sister there. She has also been theatre and ward sister and night superintendent at Queen Mary's Hospital, Carshalton, and night and ward sister at the Victoria Hospital, Bournemouth.

General Hospital, Cheltenham.—Miss D. M. Hubbard, Q.A.I.M.N.S.R., has been appointed sister. She was trained at Addenbrooke's Hospital, Cambridge, and has been sister at the General Hospital, Malvern.

**NOTE.—Particulars of Appointments for insertion in this column will be welcomed.**

### The Book Market.

LIST OF NEW BOOKS RECEIVED FROM THE FOLLOWING PUBLISHERS:—

Geo. Allen and Unwin, Ltd.: "Three Clinical Studies in Tuberculosis Predisposition." By W. C. Rivers, M.R.C.S., L.R.C.P. 12s. 6d. net.

The Scientific Press, Ltd.: "Model Answers to Questions on Nursing." By Hector Macphail. 3s. 6d. net. "Human Temperaments." By Chas. Mercier, M.D., F.R.C.P. 1s. 3d. net.

Methuen: "What Every Masseuse Should Know." By Vera Waddington. 1s. 6d. net.

W. Collins, Sons and Co., Ltd.: "Marching on Tanga." By Capt. E. Brett Young. 6s. net.

Sampson Low, Marston and Co., Ltd.: "Low's Handbook to the Charities of London." 1917.

John Wiley and Sons, Inc.: "The Pasteurisation of Milk from the Practical Viewpoint." By Charles A. Kilbourne. 6s. net.

### Papers, Pamphlets, etc.

Report on the Health of the City of Liverpool during 1916. By the Medical Officer of Health.

Annual Report (for 1916) on the Health of the City of Coventry. By the Medical Officer of Health and School Medical Officer.

### Founder's Day at the National Hospital.

Founder's Day will be celebrated at the National Hospital for the Paralysed and Epileptic, Queen Square, W.C. 1, on Friday, November 2.

